# A Chinese herbal decoction, Jian-Pi-Yi-Shen, regulates the expressions of erythropoietin and pro-inflammatory cytokines in cultured cells

**DOI:** 10.1186/s12906-018-2146-4

**Published:** 2018-04-03

**Authors:** Jianping Chen, Amy G. W. Gong, Xiaoyan Liu, Zhonggui Li, Airong Qi, Tina T. X. Dong, Tiegang Yi, Karl W. K. Tsim, Shunmin Li

**Affiliations:** 1 Shenzhen Key Laboratory of Hospital Chinese Medicine Preparation, Shenzhen Traditional Chinese Medicine Hospital, Guangzhou University of Chinese Medicine, Shenzhen, Guangdong China; 2 Division of Life Science and Center for Chinese Medicine, The Hong Kong University of Science and Technology, Hong Kong, China; 3 Department of Nephrology, Shenzhen Traditional Chinese Medicine Hospital, Guangzhou University of Chinese Medicine, Shenzhen, Guangdong China

**Keywords:** Herbal medicine, Erythropoietin, Pro-inflammatory cytokines

## Abstract

**Background:**

A Chinese herbal formula, namely Jian-Pi-Yi-Shen (JPYS), has been clinically prescribed for patients with chronic kidney disease associated-anemia, and which can improve the patient’s immunological system. However, the mechanisms of JPYS involved in anemia and immune response have not been investigated. To study the role of JPYS in regulating hematopoietic and immunological functions, we investigated its activities on the expressions of erythropoietin and pro-inflammatory cytokines in cultured cells.

**Methods:**

The standardized herbal extracts of JPYS (0–30 μg/ml) were applied onto cultured cells for 24–48 h. Total RNA was collected from the treated cells and subjected to real-time quantitative PCR analysis. Cultured HEK293T cells, transfected with a construct composed of hypoxia response element tagged with a luciferase gene, i.e. pHRE-Luc, were treated with JPYS extracts (1–30 μg/ml) for 24 h. The cell lysates were subjected to luciferase assay.

**Results:**

The treatment with JPYS extract onto cultured HEK293T cells induced erythropoietin expression in a dose-dependent manner, having the highest response by ~ 50% of increase. In parallel, application of JPYS extract for 24 h stimulated expressions of interleukin (IL)-1β, IL-6, and tumor necrosis factor (TNF)-α in cultured RAW 264.7 macrophages. In contrast, the pretreatment with JPYS extract suppressed expressions of IL-1β, IL-6, and TNF-α in lipopolysaccharide-induced macrophages.

**Conclusions:**

These results confirmed the hematopoietic function of JPYS in regulating erythropoietin expression, as well as the bidirectional immune-modulatory roles of JPYS by regulating the expression of pro-inflammatory cytokines in cultures.

## Background

Jian-Pi-Yi-Shen (JPYS), a Chinese herbal decoction, is comprised of eight medicinal herbs, i.e., Astragali Radix, Atractylodis Macrocephalae Rhizoma, Dioscoreae Rhizoma, Cistanches Herba, Amomi Fructus Rotundus, Salviae Miltiorrhizae Radix et Rhizoma, Rhei Radix et Rhizoma, Glycyrrhizae Radix et Rhizoma Praeparata cum Melle. For over 20 years, JPYS has been clinically prescribed for patients with chronic kidney disease (CKD) associated-anemia, as it is believed to possess the efficacies of fortifying the spleen, tonifying the kidney, activating blood and resolving stasis. The most known cause of CKD anemia is an insufficient erythropoietin (EPO) production [1]. Recombinant human erythropoietin (rHuEPO) treatment has been employed to correct CKD associated-anemia as to improve the quality of patients’ life. However, the potential side effects in cardiovascular system and weak response in some CKD-associated patients have restricted the usage of rHuEPO [2]. Apart from aforesaid treatments, a number of patients also may choose alternative therapeutic approaches, such as traditional Chinese medicine (TCM). Previous pharmacological studied also showed that JPYS could increase the hemoglobin level in the nephrectomised rats [3]. In parallel, clinical studies demonstrated that JPYS could regulate the level of interleukin (IL)-1, IL-2 and IL-6, as to improve patient’s immunological system [4]. These studies indicated the trophic role of JPYS in blood nourishment and immunological system. However, the molecular mechanism of JPYS in nourishing blood and immune response remains unclear.

In recent findings, CKD anemia might result from a defective hypoxic signaling rather than the lack of EPO-stimulating cells to synthesize EPO [5]. Bernhardt et al. (2010) reported that the hypoxia-inducible factor (HIF) signaling could increase EPO production in CKD patients [6]. We therefore speculated that JPYS might stimulate EPO expression via HIF signaling in achieving for the treatment of CKD anemia. Besides, the pro-inflammatory cytokines, produced by macrophages, play a vital role in the early stage of immune response [7]. Here, the transcriptional expression of EPO was analyzed in cultured kidney fibroblasts, HEK293T cells, and the regulatory role of JPYS in pro-inflammatory cytokine expression in RAW 264.7 macorphages was investigated.

## Methods

### Chemical and reagent

Echinacoside (Lot No. 111670–201,304), salvianolic acid B (Lot No. 110821–201,313), rhein (Lot No. 110757–200,206) were purchased from National Institutes for Food and Drug Control (Beijing, China). The purities of all marker chemicals were determined to be no less than 98% by normalization of peak areas, as revealed by HPLC-DAD. HPLC grade acetonitrile was purchased from Merck (Darmstadt, Germany), and ultrapure water was prepared using a Milli-Q purification system (Molsheim, France). Other reagents used here were of analytical grade. Reagents for cell cultures were purchased from Invitrogen Technologies (Carlsbad, CA). Mineral oil (hypoxia mimicry), lipopolysaccharide (LPS, a pro-inflammatory stimulus from *Escherichia coli*) and dexamethasone (an anti-inflammatory agent) were purchased from Sigma (St. Louis, MO) [8, 9].

### Plant materials and preparation of JPYS extract

Plant materials: Astragali Radix (Lot. 150,621; roots of *Astragalus membranaceus* (Fisch.) Bge. var. *mongholicus* (Bge.) Hsiao), Atractylodis Macrocephalae Rhizoma (Lot. 141,230; rhizomes of *Atractylodes macrocephala* Koidz.), Dioscoreae Rhizoma (Lot. 150,615; rhizomes of *Dioscorea opposite* Thunb.), Cistanches Herba (Lot. 150,621; herbs of *Cistanche deserticola* Y.C. Ma), Amomi Fructus Rotundus (Lot. 150,617; fruits of *Amomum kravanh* Pierre ex Gagnep.), Salviae Miltiorrhizae Radix et Rhizoma (Lot. 150,626; roots and rhizomes of *Salvia miltiorrhiza* Bge.), Rhei Radix et Rhizoma (Lot. 150,104; roots and rhizomes of *Rheum palmatum* L.), Glycyrrhizae Radix et Rhizoma Praeparata cum Melle (Lot. 150,615; roots and rhizomes of *Glycyrrhiza uralensis* Fisch.) were purchase from Shenzhen Huahui Pharmaceutical Co., Ltd. The plant materials were authenticated by Dr. Tina Dong based on their morphological characteristics. The voucher specimens were kept at Pharmaceutical Department, Shenzhen Traditional Chinese Medicine Hospital with number 2010015Z, 2010024ZZ, 2010037Z, 2040056Z, 202086Z, 2010006Z, 2010040Z and 2010008ZZ, respectively. Assurance of quality control for all the materials was validated according to the Chinese Pharmacopeia (China Pharmacopoeia Committee, 2015).

Astragali Radix (30 g), Atractylodis Macrocephalae Rhizoma (10 g), Dioscoreae Rhizoma (30 g), Cistanches Herba (10 g), Amomi Fructus Rotundus (10 g), Salviae Miltiorrhizae Radix et Rhizoma (15 g), Rhei Radix et Rhizoma (10 g), Glycyrrhizae Radix et Rhizoma Praeparata cum Melle (6 g) were weighed and extracted in boiling water (1.2 L) twice for 1 h. After centrifugation (13,000 rpm, 10 min), the supernatant was stored at 4 °C and filtered through a 0.22 μm filter (Millipore Ireland Ltd., Ireland) before injection into HPLC system for analysis. For biological analysis, the extract was dried under reduced pressure to powder, and it was stored at − 80 °C. Before the treatment, the powder was re-dissolved with Milli-Q water and vortexed at room temperature to a concentration of 100 mg/mL of jujube extract. The solution was filtered through a 0.22 μm filter before application onto cultured cells.

### Chromatographic conditions and instrumentation

Validation HPLC method, as described in previous study [10], was performed on a Shimadzu (Kyoto, Japan) LC-20AT system, which was equipped with a degasser, a binary pump, an autosampler and a diode array detector. The herbal extract was separated on Inertsil ODS-SP (150 mm × 4.6 mm, 5 μm) column. The mobile phase was composed of 0.1% formic acid (A) and acetonitrile (B) using the following gradient program: 0–5 min, 90% A; 5–10 min, 90%–80% A; 10–30 min, 80%–60% A; 30–40 min, 90% A; the flow rate was 1.0 ml/min; the injection volume was 10 μL; the wavelength was 260 nm. A pre-equilibration period of 10 min was used between each run. The content of tested markers was calculated using their calibration curve with regarding to the dilution factor and expressed as microgram per gram of dried extract weight.

### Cell culture and treatment

Human embryonic kidney fibroblast (HEK) 293 T cells were obtained from American Type Culture Collection (ATCC, Manassas, VA). HEK293T T cells were cultured in Dulbecco’s modified Eagle’s medium (DMEM), supplemented with 10% fetal bovine serum, 100 μg/mL streptomycin, and 10% fetal bovine serum. Before plating, cells were rinsed with 5 mL phosphate buffered saline containing 1 mM EDTA twice, and followed by using 1 mL trypsin/EDTA solution to detach the cells. For EPO mRNA analysis, cultured HEK293T cells (5 × 10^4^ cells/ml) were treated with JPYS extracts (1–30 μg/mL) for 48 h. For luciferase assay, cultured HEK293T cells, transfected with pHRE-Luc, were treated with JPYS extracts (1–30 μg/ml) for 24 h. The mineral oil layering (oil: medium = 2: 1) served as a positive control. The untreated culture was served as a blank control. RAW 264.7 murine macrophages from American Type Culture Collection (ATCC, Manassas, VA) were cultured in Dulbecco’s modified Eagles medium (DMEM) supplemented with 100 IU/mL penicillin, 100 μg/mL streptomycin, and 10% heat in-active fetal bovine serum (FBS). Cells were incubated at 37 °C in a water-saturated 5% CO_2_ incubator. Before plating, cells were dislodged from plate with a cell scraper (Corning Incorporated; Corning, NY). For cytokine upregulation, cultured RAW 264.7 macrophages were treated with JPYS extract at various concentrations (0–30 μg/ml) or LPS (1 μg/ml) for 24 h. For cytokine downregulation, cultured RAW 264.7 macrophages were pre-treated with dexamethasone (Dex.; 10 μM; a positive control of suppressor) or JPYS extract at various concentrations (0–30 μg/ml) for 3 h. The untreated culture was employed as a blank control. Then, LPS (1.0 μg/ml) was applied onto the cultures for 24 h.

### Quantitative real-time PCR

For the analyses of gene expressions in HEK293T cells and RAW 264.7 cells, the cultures were treated with JPYS extract. After the treatment, total RNA was isolated by RNAzol reagent (Molecular Research Center, Cincinnati, OH), and reverse transcribed into cDNA according to the manufacturer’s instructions (Invitrogen). Real-time PCR was performed by using FastStart Universal SYBR Green Master (ROX) according to the manufacturer’s instructions (Roche Applied Science, Mannheim, Germany). The primers were: 5′- ACT TTC CGC AAA CTC TTC CG-3′ (sense primer, S) and 5′- TGA ATG CTT CCT GCT CTG GG-3′ (anti-sense primer, AS) for human EPO (330 bp; NM_000799.2); 5′- GCT TTA ACT TTG CTG GCC CCA GC-3′ (S) and 5′- GCA GGG TCA GCA CTA CTT CGA AG-3′ (AS) for human HIF-1α (221 bp; NM_001530.3); 5′- AAATAC CTG TGG CCT TG-3′ (S) and 5′- TTA GGA AGA CAC GGA TTC-3′ (AS) for murine IL-1β (296 bp; NM_008361); 5′- GGA GTA CCA TAG CTA CCT GG-3′ (S) and 5′- CTA GGT TTG CCG AGT AGA TC-3′ (AS) for murine IL-6 (283 bp; NM_031168); 5′- AGT GAC AAG CCT GTA GCC-3′ (S) and 5′- AGG TTG ACT TTC TCC TGG-3′ (AS) for murine TNF-α (251 bp; NM_013693). Glyceraldehyde 3 -phosphate dehydrogenase (GAPDH), a house-keeping gene, was used as an internal control in all cases, and its primer sequences were 5′- AAC GGA TTT GGC CGT ATT GG-3′ (S) and 5′- CTT CCC GTT CAG CTC TGG G-3′ (AS) (657 bp; NR_0215885). SYBR green signal was detected by ABI 7500 Fast Real-Time PCR system (Applied Biosystems, Foster City, CA). Transcript levels were quantified by using ΔΔCt value method [11], where the values of target genes were normalized by GAPDH in the same sample at first before comparison. PCR products were analyzed by gel electrophoresis and melting curve analysis, as to confirm the specific amplification.

### DNA transfection and luciferase assay

Six hypoxia responsive elements (HRE: 5’-TCG AGG CCC TAC GTG CTG TCT CAC ACA GCC TGT CTG ACG-3′) were synthesized, concatemerized and then cloned in tandem (head-to-tail orientation) into pBI-GL vectors (BD Biosciences Clontech, San Jose, CA) that has a downstream reporter of firefly luciferase gene: this vector was named as pHRE-Luc [12, 13]. Cultured HEK293T cells were transiently transfected with pHRE-Luc by the calcium phosphate precipitation method. The transfection efficient was over 80%, as determined by another control plasmid of having a *β*-galactosidase, under a cytomergalovirus enhancer promoter. After 24 h of transfection, the luciferase assay was performed by a commercial kit (Tropix Inc., Bedford, MA). In brief, cultures were lysed by a buffer containing 100 mM potassium phosphate buffer (pH = 7.8), 0.2% Triton X-100 and 1 mM dithiothreitol. The luminescent reaction was quantified in a Tropix TR717TM Microplate Luminometer, and the activity was expressed as absorbance (up to 560 nm) per mg of protein.

### Other assays

Protein concentrations were measured by Bradford’s method with a kit from Bio-Rad Laboratories (Hercules, CA). Individual data was expressed as Mean ± standard deviation (SD). Statistical analyses were performed using one-way ANOVA (version 13.0, SPSS). Statistically significant changes were classified as significant (*) where *p <* 0.05, more significant (**) where *p <* 0.01 and highly significant (***) where *p* < 0.001.

## Results

### Preparation of standardized JPYS

In order to chemically standardize JPYS extract, we employed the established HPLC fingerprint and quantification method to reveal its HPLC profile and quantify the main ingredients. A typical HPLC fingerprint at absorbance of 260 nm was developed for JPYS extract [10]: the fingerprint was served as an index for identification of JPYS. By using the respective individual standard, the chemical markers were identified from the extract, e.g. sodium danshensu, echinacoside, acteoside, calycosin 7-O-β-glucoside, salvianolic acid B, formononetin and rhein. Moreover, a rapid HPLC-DAD method was developed to simultaneously quantify 3 main ingredients in JPYS extract. These chemical markers included: echinacoside, salvianolic acid B and rhein. A minimal requirement for the amounts of echinacoside, salvianolic acid B and rhein should be no less than 1.2 mg/g, 5.7 mg/g and 0.2 mg/g of the dried extract. The yield of extraction was ~ 32.59 ± 1.1% (*w*/w, Mean ± SD, *n* = 3). The extract being used here reached the aforesaid requirements, which guaranteed the repeatability of biological results.

### JPYS induces the expression of EPO

**Fig. 1:**
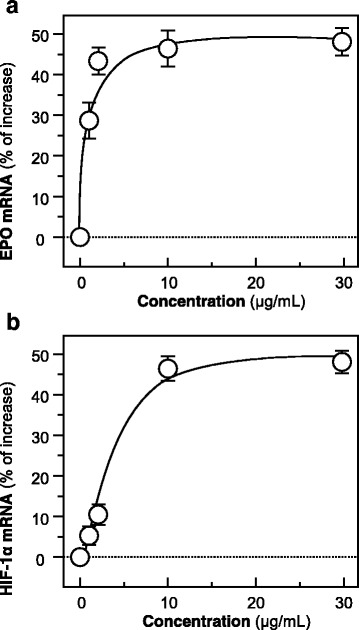
JPYS stimulates the mRNA expressions of EPO and HIF-1α. **a** Cultured HEK293T cells (5 × 10^4^ cells/ml) were applied with JPYS extracts (1–30 μg/mL) for 48 h. The level of EPO mRNA was detected by real-time PCR. **b** Cultured HEK293T cells were treated with JPYS extracts as in (**a**).The level of HIF-1α mRNA was detected by real-time PCR. GAPDH was used as an internal control for normalization. The mineral oil layering (oil: medium = 2: 1) served as a positive control, with the response about 80% and 150% of increase for expressions of EPO and HIF-1α mRNAs, respectively. Values are expressed as % of increase to basal reading (untreated culture), and in Mean ± SD, where *n* = 3, each with triplicate samples. Statistical comparison was made with the control; * *p* < 0.05; ** *p* < 0.01

To investigate the effect of JPYS, the extract of JPYS was applied onto cultured HEK293T cells for 48 h. The concentrations chosen in this experiment did not show cytotoxicity on the cultures. Total RNA was collected from the treated cells and subjected to real-time quantitative PCR analysis by using specific primers flanking the EPO mRNA. The treatment with JPYS at various concentrations (0–30 μg/ml) induced the amount of EPO mRNA levels, and the effect of which was demonstrated to be dose-dependent, having the highest response by ~ 50% of increase (Fig. 1a). In order to reveal the regulatory mechanism of hypoxia inducible factor (HIF) in JPYS-induced HRE activation, the HIF signaling cascade involving in expression of HIF-1α was analyzed. Here, the amount of mRNAs encoding HIF-1α was determined in JPYS-treated HEK293T cells. The application of JPYS at various concentrations (0–30 μg/ml) stimulated the amount of HIF-1α mRNA level, and the effect was shown to be dose-dependent, having the highest response by ~ 50% of increase (Fig. 1b). The mineral oil layering, mimicking hypoxia condition, served as a positive control.

**Fig. 2:**
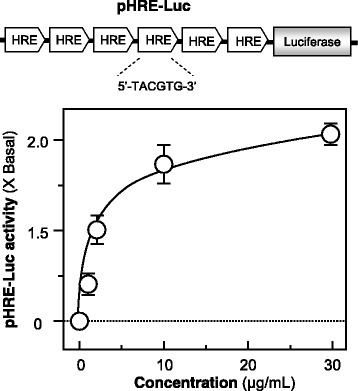
JPYS induces HRE-mediated transcriptional activity. A luciferase-reporter containing six HREs and a downstream luciferase-reporter gene, namely as pHRE-Luc, was used as a study tool (upper panel). Cultured HEK293T cells, transfected with pHRE-Luc, were treated with JPYS extracts (1–30 μg/ml) for 24 h. The cell lysates were subjected to luciferase assay. The mineral oil layering (oil: medium = 2: 1) served as a positive control, with the response about 70% of increase. Values are expressed as the fold of increase to basal reading (untreated culture), and they are in Mean ± SD, where *n* = 3, each with triplicate samples. Statistical comparison was made with the control; * *p* < 0.05; ** *p* < 0.01; *** *p* < 0.001

The activation of hypoxia-mediated signaling pathway is an inducer for EPO gene expression [14]. To reveal the transcriptional activity of HRE, a luciferase-reporter construct (pHRE-Luc), containing six HREs derived from the enhancer of EPO gene and tagged upstream of a luciferase gene was transfected into cultured HEK293T cells (Fig. 2, upper panel). To investigate the activation of HRE-mediated pathway, JPYS extracts at various concentrations were applied onto pHRE-Luc-expressed HEK293T cells. Here, JPYS extracts activated the HRE-mediated transcriptional activity in a dose-dependent manner, having the highest response by ~ 2-fold of increase under treatment of 30 μg/ml (Fig. 2, lower panel). The mineral oil layering served as a positive control.

### JPYS regulates the expression of pro-inflammatory cytokines

**Fig. 3:**
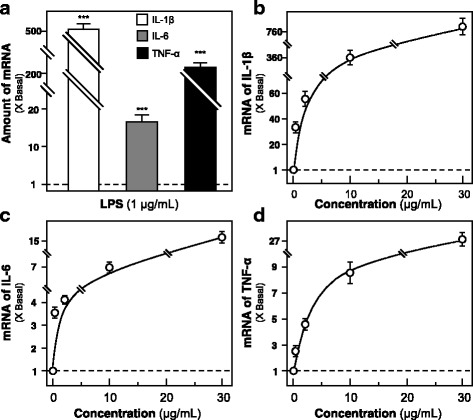
JPYS induces the expressions of pro-inflammatory cytokines in cultured RAW 264.7 cells. **a** Cultured RAW 264.7 macrophages were treated with LPS (1 μg/ml) for 24 h. LPS could robustly induce pro-inflammatory cytokine expression and which was employed as a positive control. **b**-**d** Cultured RAW 264.7 macrophages were treated with JPYS extract at various concentrations (0–30 μg/ml) for 24 h. The mRNA levels of pro-inflammatory cytokines (IL-1β, IL-6 and TNF-α) were revealed by real time PCR. GAPDH mRNA served as an internal control for normalization. Data are expressed as the fold of increase to basal reading (untreated cultures), and in Mean ± SD, where *n* = 3, each with triplicate samples. Statistical comparison was made with the control; *** *p* < 0.001

**Fig. 4:**
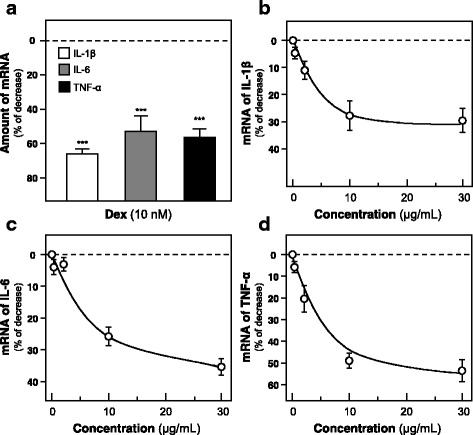
JPYS suppresses the LPS-induced expression of pro-inflammatory cytokines in cultured macrophages. Cultured RAW 264.7 macrophages were pre-treated with Dexamethasone (Dex.; 10 μM; positive control of suppressor) (**a**) or JPYS extract (**b**-**d**) at various concentrations (0–30 μg/ml) for 3 h. Then, LPS (1.0 μg/ml) was applied onto the cultures for 24 h to mimic the chronic inflammation. The mRNA levels of pro-inflammatory cytokines IL-1β, IL-6 and TNF-α were reveal by real time PCR. GAPDH was served as an internal control for normalization. Values are expressed as % of the LPS-induced activation, and in Mean ± SD, where *n* = 3, each with triplicate samples. Statistical comparison was made with the control; * *p* < 0.05; ** *p* < 0.01; *** *p* < 0.001

In order to reveal the immune-modulatory activities of JPYS at cellular level, macrophage activation involving induction of pro-inflammatory cytokines, e.g. IL-1β, IL-6 and TNF-α, were studied. JPYS extracts at various concentrations (0–30 μg/ml) were applied onto cultured RAW 264.7 cell for 24 h. The extract induced the transcriptional expression of IL-1 β, IL-6 and TNF-α in a concentration-dependent manner, having the highest response at ~ 700-fold, ~ 15-fold and 20-fold under the treatment of JPYS extract at 30 μg/ml (Fig. 3). Furthermore, the effect of JPYS in suppressing the expression of pro-inflammatory cytokines under the LPS-challenged cell model. LPS (1 μg/ml) was applied onto the cultured macrophages, as to mimic the excessive activation. Dexamethasone served as a positive control. The pre-treatment with JPYS extract dose-dependently suppressed the expressions of mRNAs encoding for IL-1β, IL-6 and TNF-α in LPS-induced RAW 264.7 cells, having the highest reduction at ~ 30%, ~ 35% and 50% under the pre-treatment of JPYS extract at 30 μg/ml (Fig. 4).

## Discussion

CKD is a worldwide health issue affecting ~ 7% of the population over the age of 30 [5]. Anemia is a very common major complication of CKD. EPO is a potent stimulator of erythropoiesis and produced mainly by kidney and liver: its production plays key role in the formation and production of red blood cell [15]. rHuEPO is commonly used to treat CKD associated-anemia. However, there are some side effects related to the usage of rHuEPO [2]. Traditional herbal medicines have been developed over thousands of years. Extensive experience and lots of clinical data for the therapy of CKD have been documented, which is very attractive for development of therapeutic drugs. Our current studies verified the hematopoietic function of JPYS by regulating EPO expression via hypoxia-inducible factor-1α in kidney cells, suggesting that JPYS could be employed as an alternative medicine for the treatment of CKD associated-anemia. In support of this notion, our previous animal studies revealed that JPYS could increase the hemoglobin level [3].

Astragali Radix was served as sovereign medicine in JPYS formula. Astragali Radix is widely used as a health supplement to reinforce the body vital energy, and also well-known to improve the hypoxia condition. Regarding to EPO production, flavonoids have been reported to induce the expression of EPO [16]. Here, we assumed that flavonoids might be one of the active ingredients in JPYS that possessed the property to stimulate the EPO expression. In agreement with this notion, Zheng et al. (2011) reported that flavonoids from Astragali Radix including formononetin, ononin, calycosin, and calycosin-7-O-β-D-glucoside were able to stimulate EPO expression in cultured cells. And this effect was involved in the regulation of HIF signaling [16]. In our current study, seven compounds were identified in JPYS extract, which were reported to be relatively higher amount in the herbs and/or possessed biological activities [17, 18]. Thus, these parameters are recommended in making a standardized JPYS extract. Indeed, some chemical markers from Astragali Radix, such as formononetin and calycosin-7-O-β-D-glucoside, were also indentified in our current HPLC analysis. In addition, there are other flavonoids within other herbs in JPYS, such as echinacoside, acteoside and liquiritin. Whether these chemicals could induce EPO production however, which needs to be further studied.

On the other hand, immune deficiency is reported to complicate with loss of renal function and contributes significantly to patients with end-stage renal disease in terms of overall mortality and morbidity [19]. Macrophages are well-known to initiate specific defense mechanisms of our immune response. The pro-inflammatory cytokines, secreted by macrophages, play a vital role in early immune system [7, 9]. IL-1β, IL-6 and TNF-α are the key pro-inflammatory cytokines playing critical roles in mediating the immune response [20]. Thus, these cytokines were being targeted in our current studies. The pro-inflammatory could activate macrophages, as the first line of defense against pathogens. Excessive activation of inflammatory cytokines, however, showed the damaging effects [9]. According to TCM theory, JPYS is able to fortify the spleen, which is referring to immune functions in modern medicine. Here, we found that JPYS exerted both pro- and anti-inflammatory activities in cultured RAW 264.7 cells. JPYS induced the expressions of pro-inflammatory cytokines, e.g. IL-1β, IL-6 and TNF-α. On the other hand, JPYS suppressed the expressions of LPS-induced cytokines. Hence, we proposed that JPYS extract possessed bi-directional immune-modulatory roles by regulating the expression of pro-inflammatory cytokines under different scenarios in macrophages. This notion is fully supported the clinical usage of JPYS for CKD patients having a complication of immune deficiency. In line with these findings, previous studies demonstrated that JPYS could regulate the level of IL-1, IL-2 and IL-6 in patients with kidney disease [4]. In addition, JPYS could improve patient’s immune response by up regulating the level of CD3+, CD4+, CD4+/CD8+ lymphocyte subgroups, as well as IL-2, and down regulating the level of CD8+, IL-1 and IL-6 [21].

## Conclusions

In conclusion, JPYS induced the expression of EPO and activated the HRE-mediated transcriptional activity, which confirmed the hematopoietic function of JPYS. Moreover, JPYS possessed bi-directional immune-modulatory roles by regulating the expression of pro-inflammatory cytokines under different scenarios in macrophages, which may support the clinical usage of JYF for CKD patients having a complication of immune deficiency.

## Abbreviations

CKD: chronic kidney disease
EPO: erythropoietin
HIF: hypoxia-inducible factor
HRE: hypoxia response element
IL: interleukin
JPYS: Jian-Pi-Yi-Shen
LPS: lipopolysaccharide
PCR: polymerase chain reaction
TCM: traditional Chinese medicine
TNF: tumor necrosis factor
